# A Rare Case of Corneal Perforation Secondary to Gonococcal Keratoconjunctivitis

**DOI:** 10.7759/cureus.74312

**Published:** 2024-11-23

**Authors:** Che Ku Hafiza Che Ku Amran, Qi Zhe Ngoo, Fadil Awis Qarni

**Affiliations:** 1 Ophthalmology and Visual Science, Universiti Sains Malaysia, Kota Bharu, MYS; 2 Ophthalmology, Universiti Sains Malaysia, Kota Bharu, MYS

**Keywords:** ceftriaxone, corneal perforation, gonococcal, keratoconjunctivitis, neisseria gonorrhoeae

## Abstract

Gonococcal keratoconjunctivitis (GKC) is an aggressive infection caused by *Neisseria gonorrhoeae*, which can cause an acute, dreadful, ulcerative keratitis resulting in blindness if left untreated. We report a rare case of bilateral GKC complicated with left eye corneal perforation. A 20-year-old male presented with bilateral eye purulent discharge associated with vision loss over the left eye for two weeks prior to presentation. Gram stain from purulent discharge showed gram-negative diplococci and cultured positive for *N. gonorrhoeae*. Left eye corneal perforation was managed with cyanoacrylate glue and a bandage contact lens. Systemic and topical antibiotic treatments were administered. Resolution was achieved with the administration of one gram of intramuscular ceftriaxone in a single dose, and the patient had no recurrences. Prompt and effective antibiotic administration in the early stage of disease contributed to an adequate recovery in this case.

## Introduction

Gonococcal keratoconjunctivitis (GKC) is a rapidly progressing infection caused by *Neisseria gonorrhoeae* [1]. This organism is able to adhere to and penetrate intact corneal epithelium as demonstrated by Tjia et al. [2]. They suggested the mechanism starts with rapid adherence of the bacteria to the epithelial cell surface mediated by pili since only piliated strains were able to adhere to the cells. Subsequently, the bacteria were then engulfed by the epithelial cells and appeared to be lying inside vacuoles within one hour after inoculation of the bacteria. After about 24 hours, the epithelium thickness was found to be considerably reduced and probably caused by a continuous desquamation of infected cells. All these processes will eventually lead to epithelial, stromal, and ulcerative keratitis, which may progress to corneal perforation [3].

Symptoms include acute pain, redness, and hyperacute profuse purulent discharge. Signs include conjunctival chemosis, globe tenderness, eyelid edema, and preauricular lymphadenopathy [4]. Untreated cases can result in severe complications such as vision loss if the bacteria penetrate further and cause corneal ulceration and scarring [4]. Ceftriaxone may be the best available antimicrobial agent as a single-dose treatment of gonococcal conjunctivitis (GC) [1]. Therefore, treatment must be initiated as soon as possible, even before the result of the culture, due to the aggressive nature of the infection [1]. Herein, we report a rare case of a patient who presented with bilateral GKC complicated with left eye corneal perforation.

## Case presentation

**Figure 1:**
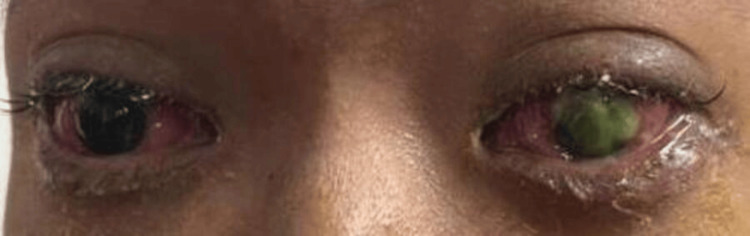
On presentation, bilateral eyes redness and swelling with excessive purulent discharge.

A 20-year-old male presented with a three-week history of bilateral eye redness and swelling associated with excessive purulent discharge, as seen in Figure 1. Subsequently, he developed vision loss in his left eye. He went to seek treatment multiple times at the nearest health clinics and was prescribed topical chloramphenicol and oral antibiotics, which he completed for a one-week duration. Unfortunately, his symptoms persisted, and thus he was referred for an ophthalmology consultation. Upon further history, he was previously well with no known medical illness and no known drug allergy. Socially, he worked as a helper at a food stall, and there was a history of illegal drug usage two years ago, which he already stopped. Furthermore, there was a significant history of recent sexual promiscuity with one partner five months prior to symptom onset. He is single and lives with his grandmother, away from his parents. Otherwise, he denied other high-risk behaviors, such as no homosexuality, no illegal drug usage, and no sexual worker involvement. He also denied any ocular trauma, no conjunctivitis contact, no previous eye disorder, no fever, and no penile or urethral discharge.

**Figure 2:**
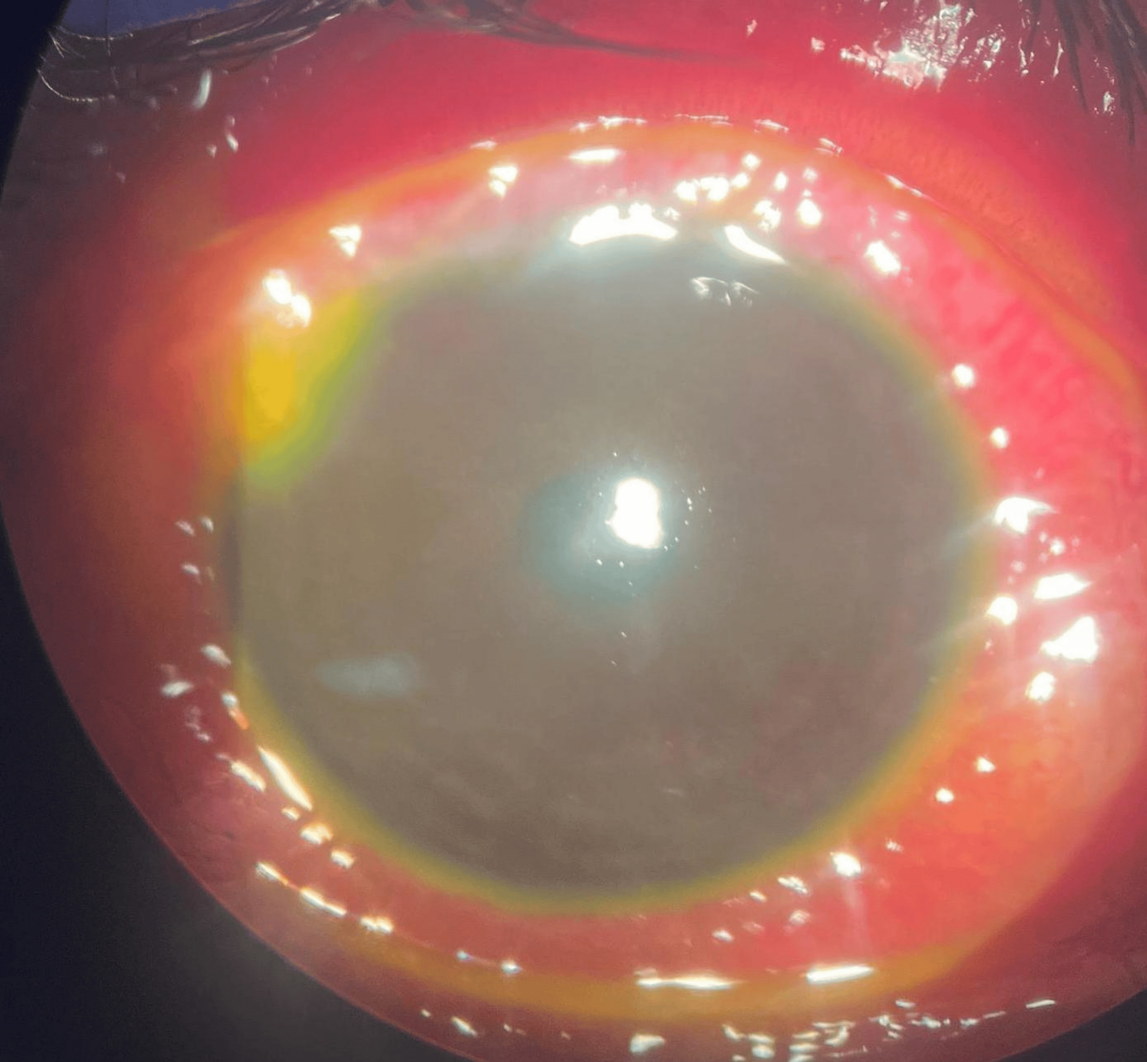
On presentation, right eye with severe conjunctival chemosis.

**Figure 3:**
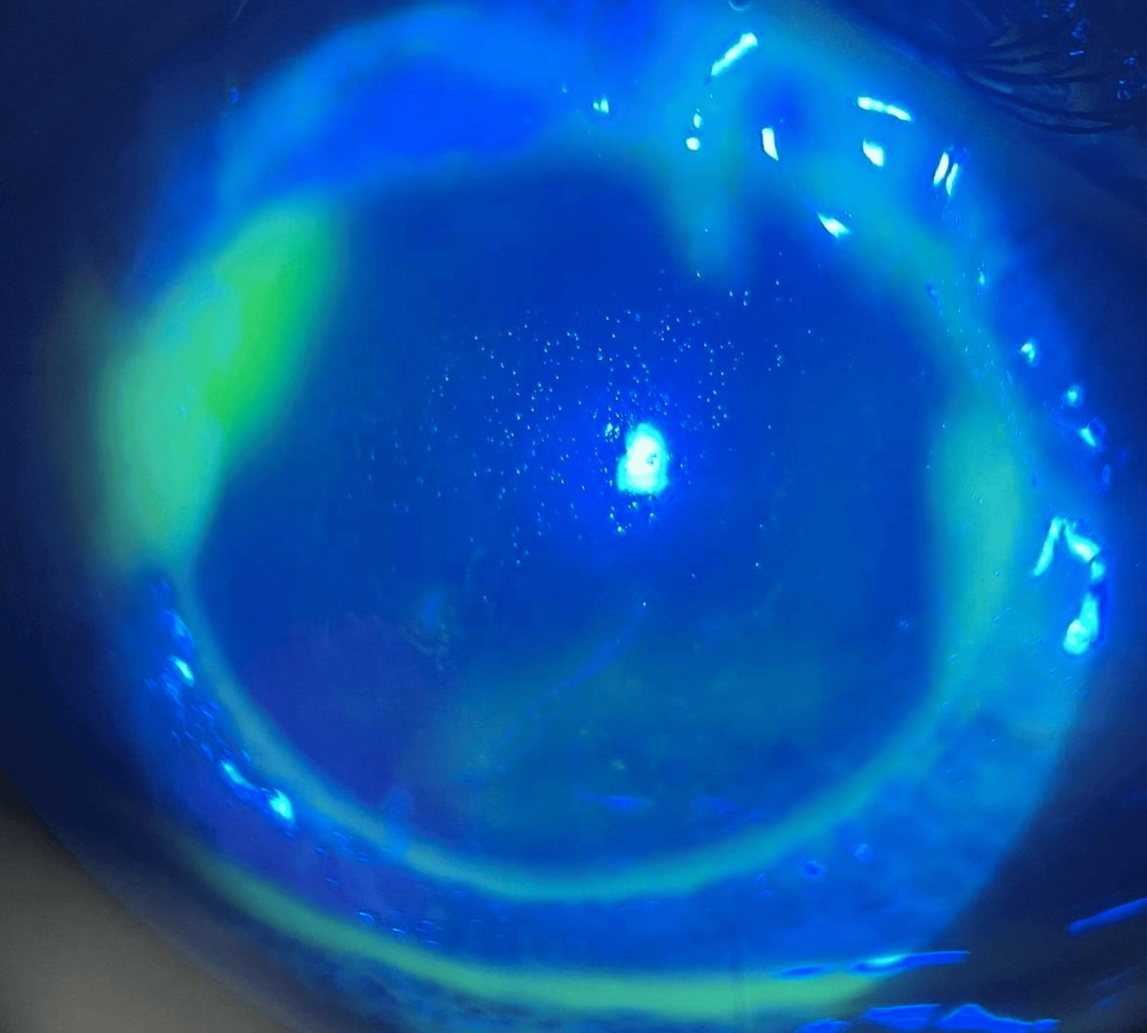
On fluorescein, right eye with epithelial defect at 11 o’clock.

Upon ocular examination, the right eye presenting acuity was 6/60 pinhole and 6/18 with negative relative afferent pupillary defect (RAPD). There was lid edema with matted lashes with purulent discharge, marked papillae, and no pseudomembrane seen. Severe conjunctival chemosis was noted 360 degrees, as seen in Figure 2. The cornea showed epithelial defect at 11 o’clock, as seen in Figure 3, with generalized superficial punctate epithelial erosion (SPEE). The anterior chamber was deep with no hypopyon. Intraocular pressure using a tonometer was 07, 08 (5%). Fundus examination was unremarkable.

**Figure 4:**
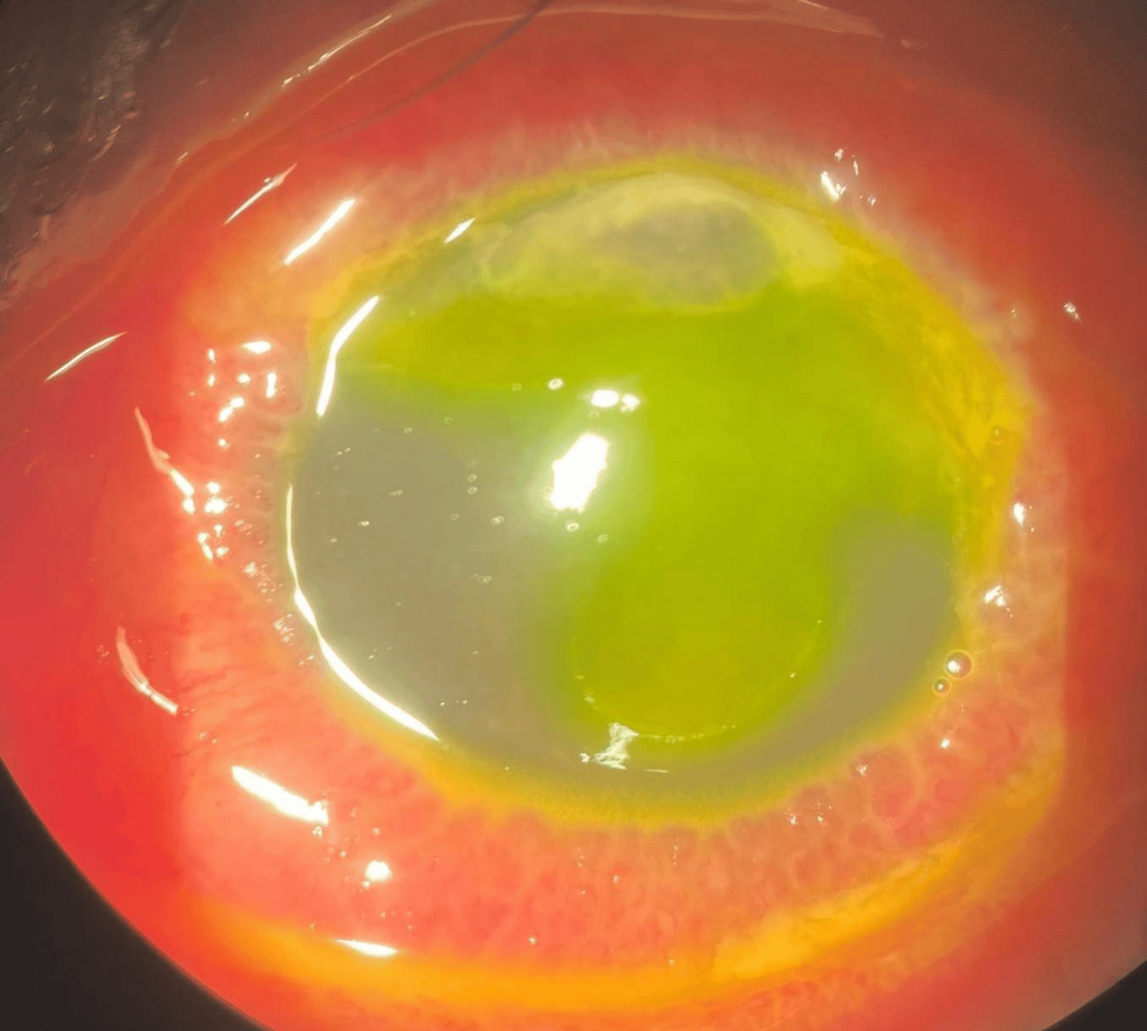
On presentation, left eye with dense infiltrate at 12 o’clock and cornea thinning.

**Figure 5:**
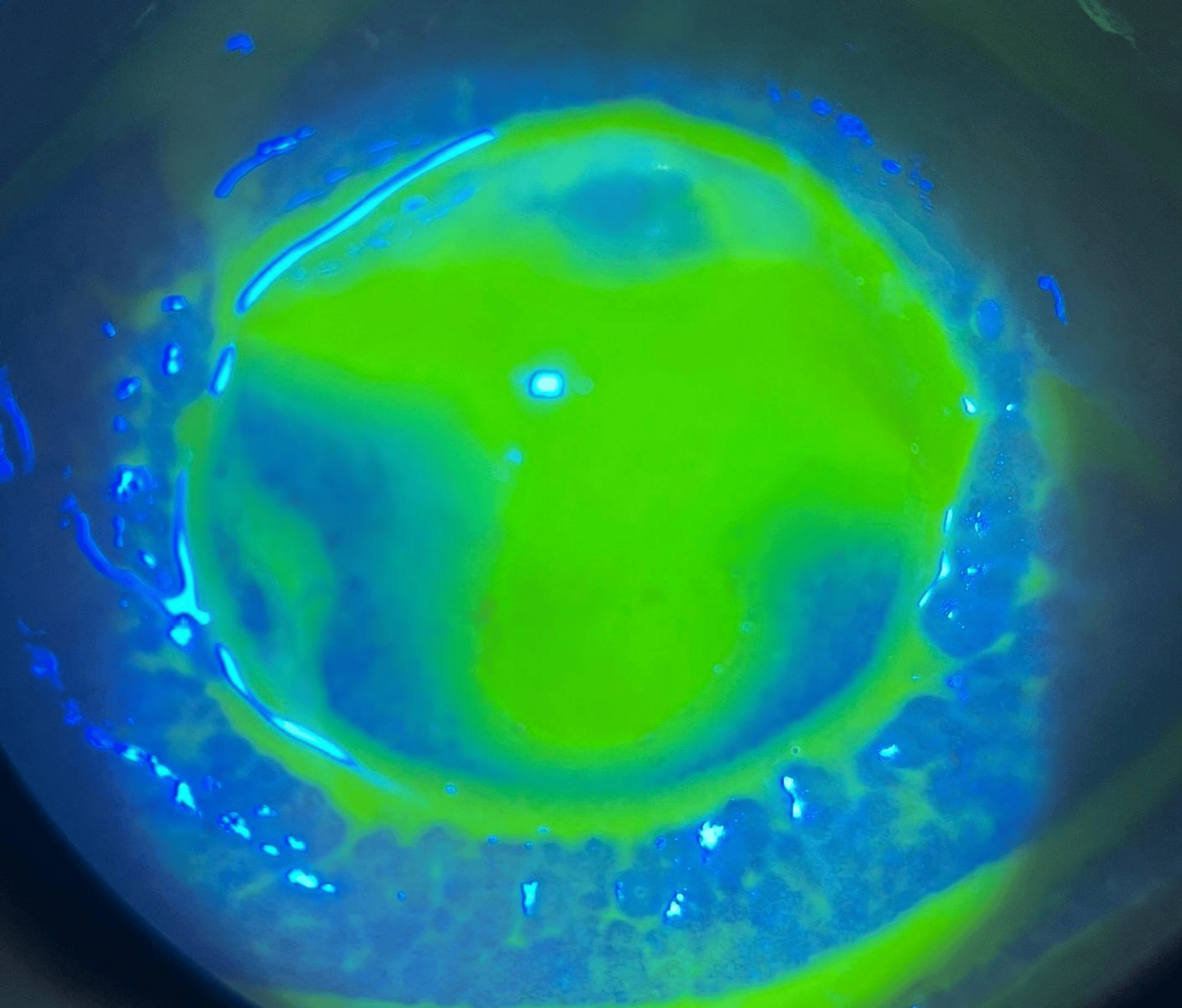
On fluorescein, left eye almost total epithelial defect with positive Seidel at 12 o’clock.

The left eye presenting acuity was hand movement (HM) with negative RAPD. There was lid edema with matted lashes with purulent discharge, marked papillae, and no pseudomembrane seen. Severe conjunctival chemosis was noted 360 degrees. The cornea showed a dense infiltrate with cornea thinning at 12 o’clock, as seen in Figure 4. Fluorescein staining revealed almost total epithelial defect with positive Seidel slow leaking at 12 o’clock where corneal thinning was noted, as seen in Figure 5. The anterior chamber was deep with no hypopyon. Intraocular pressure using a tonometer was 07, 05 (5%). Fundus examination was hazy; however, a B-scan ultrasound showed clear vitreous, and no loculations were seen.

**Figure 6:**
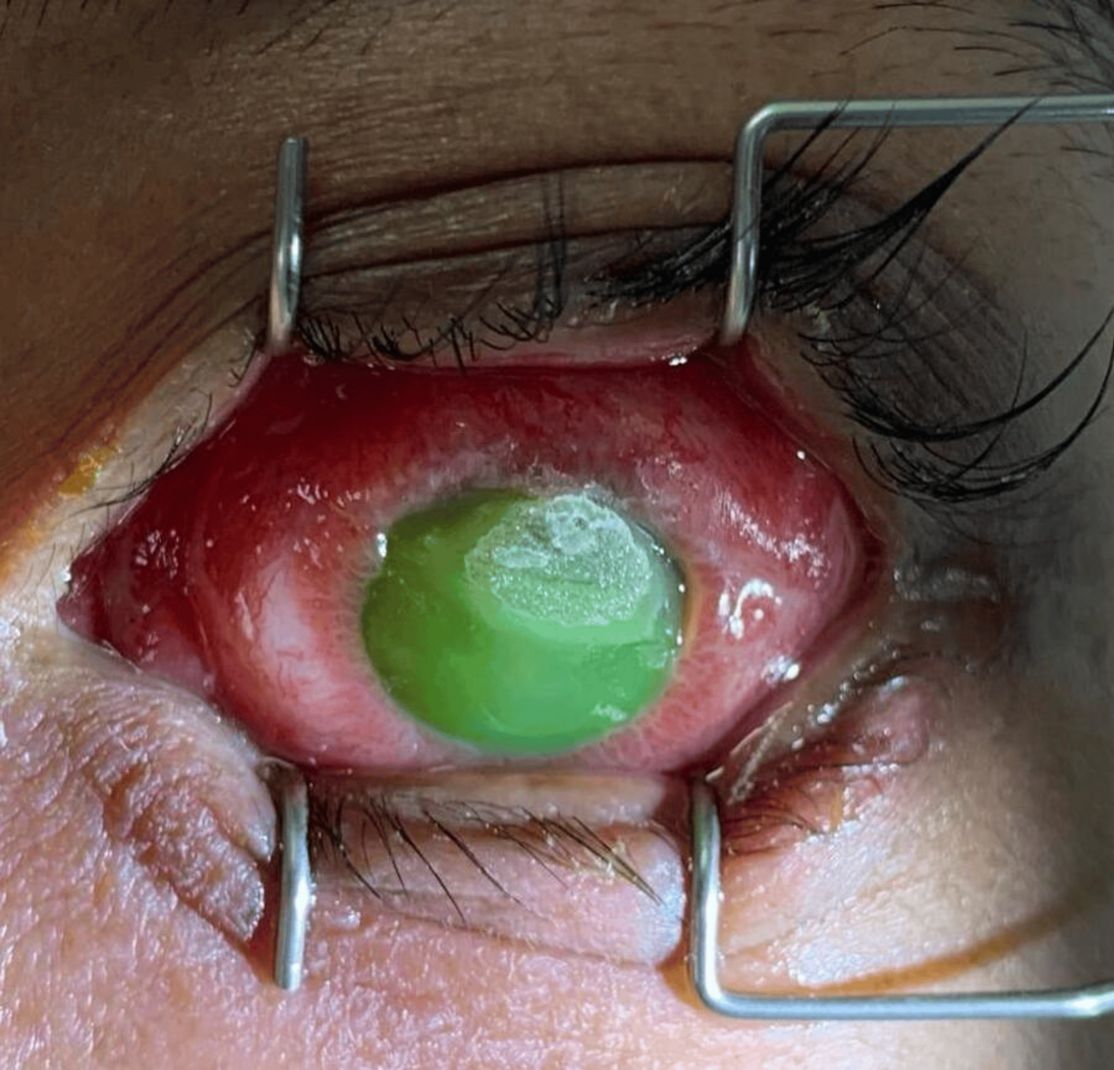
Left eye post cyanoacrylate glue over 12 o’clock perforation.

Systemic examination revealed no urethral discharge, normal penile and testicular, no ulcers or chancroid, and no inguinal lymphadenopathy. Other than borderline tachycardia, the patient remained afebrile throughout admission. A bilateral conjunctival eye swab taken revealed Gram-negative diplococci on gram staining and cultured positive for *N. gonorrhoeae*, which was sensitive to ceftriaxone. Left eye corneal scrapping culture and sensitivity (C&S) revealed no growth. A complete blood count revealed elevated white cell count with predominantly neutrophils. The patient screened negative for human immunodeficiency virus (HIV), hepatitis B, hepatitis C, and syphilis. The infectious disease (ID) team was consulted and 1 gram intramuscular (IM) ceftriaxone dose was given. The corneal perforation was managed with cyanoacrylate glue and bandage contact lens as it was a small perforation of less than 3 mm, as seen in Figure 6.

**Figure 7:**
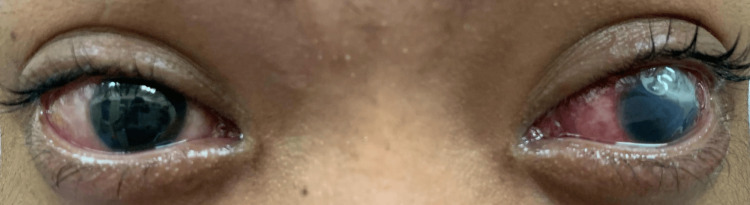
Two weeks on treatment with improved redness and swelling.

Subsequently, the patient was started on intensive topical antibiotics of 5% ceftazidime and 0.9% gentamicin eye drops with a loading dose, then hourly around the clock. He was also started on oral doxycycline 100 mg once daily and oral vitamin C 1 gram once daily. By one week of treatment, the infection was responding to treatment, evidenced by improvements in the redness and swelling, as seen in Figure 7. The visiting corneal team was consulted and planned to taper topical antibiotics first as the corneal perforation was stable with glue.

**Figure 8:**
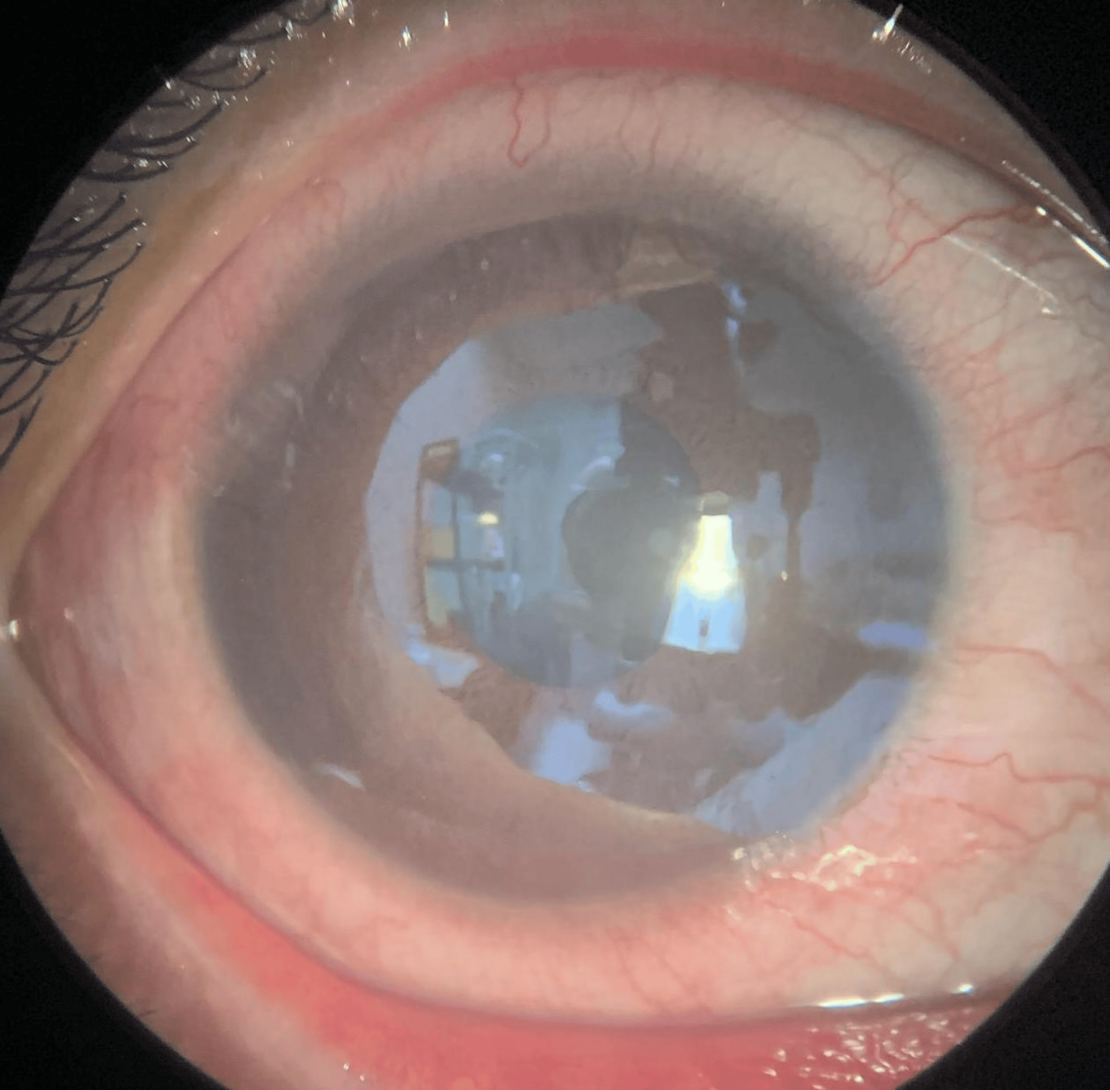
Right eye two months on treatment showed resolution signs.

**Figure 9:**
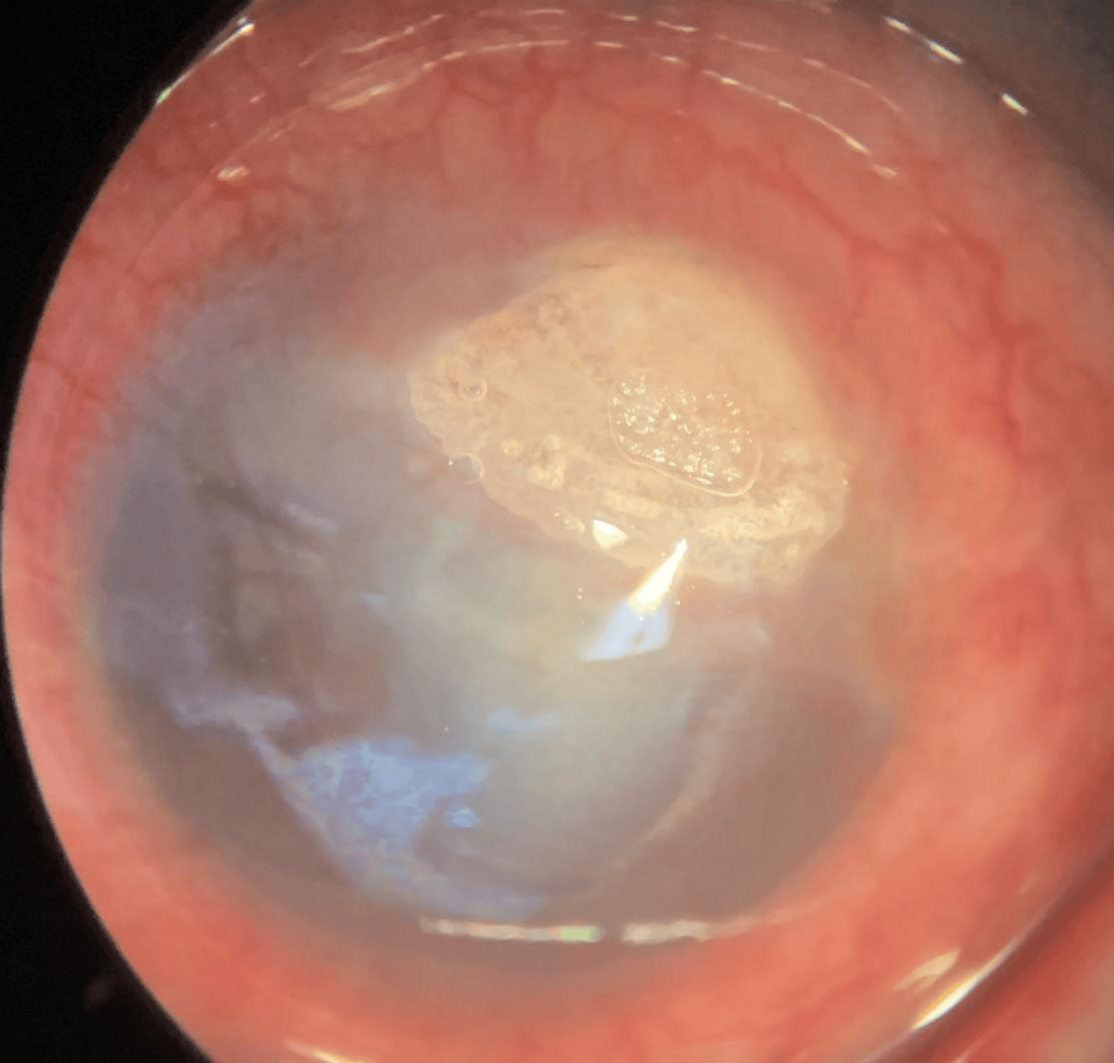
Left eye two months on treatment showed resolution signs with stable corneal glue.

The patient was followed up until two months with marked improvement over the right eye with visual acuity 6/9 and resolution of signs, as seen in Figure 8. There were no signs of complications over the right eye, such as a cornea scar or dry eyes, and the fundus was normal. The left eye showed a slight improvement in visual acuity of 1/60 with stable corneal glue, as seen in Figure 9. There were no signs of recurrent, worsening infection or endophthalmitis. However, the patient subsequently defaulted on our follow-up.

## Discussion

GC was predominantly a disease of neonates and was considered rare in adults [5]. However, a study in Ireland suggests that in recent years, the prevalence of GC has increased in young adults with male predominance [6], such as in our patient.

GKC typically occurs in males, especially with promiscuous lifestyles, by direct inoculation with infected body fluids, and it is usually unilateral [1], which was rather different in our patient, who had bilateral eye involvements.

The incubation period for gonococcal ocular infection usually ranges from 3 to 19 days, and the urethral symptoms precede the ocular symptoms from one to several weeks [7]. In comparison, our case had a relatively long incubation period, which was more than a month, and there were no preceding urethral symptoms.

Typical ocular symptoms include hyperacute, profuse, purulent discharge associated with pain and redness. Whereas ocular signs include conjunctival chemosis, globe tenderness, eyelid edema, and preauricular lymphadenopathy [4].

Untreated cases can result in severe complications such as vision loss if the bacteria penetrate further and cause corneal ulceration and scarring [4]. Therefore, the Centre for Disease Control (CDC) recommends treatment with ceftriaxone 1 g intramuscularly in a single dose [8], which results in satisfactory resolution in our patient.

Apart from systemic antibiotics, saline eyewash and topical antibiotics have been recommended as adjunctive therapy rather than essential for therapeutic success in adults [9]. Nevertheless, aggressive topical antibiotics of 5% ceftazidime and 0.9% gentamicin hourly around the clock were initiated in view of the severe condition that the patient presented to us.

The patient was also started on oral doxycycline 100 mg once daily to prevent the activity of collagenase released from destructive corneal lesions [10] and to treat possible coexistent chlamydial infection with a false negative response by laboratory test [11], as suggested by the dermatology team.

## Conclusions

We presented a case of bilateral GKC complicated with left eye cornea perforation. This case highlights the importance of complete history taking, including sexual history, which translates into early recognition and treatment to prevent complications. Unfortunately, in our case, the patient presented late with complications of corneal perforation. Therefore, a high index of suspicion during history taking is crucial, especially in young, sexually active patients, as they are among the high-risk groups contracting this illness. Timely diagnosis and prompt treatment may eventually avert both sight- and life-threatening complications related to this ID.
